# Effects of premolar extraction and orthodontic treatment in adolescents - a retrospective cephalometric study

**DOI:** 10.1080/00016357.2023.2267145

**Published:** 2024-03-26

**Authors:** Maria Ekstam, Mikael Sonesson, Kristina Hellén-Halme

**Affiliations:** a Department of Oral and Maxillofacial Radiology, Malmö University, Malmö, Sweden; b Department of Orthodontics, Malmö University, Malmö, Sweden

**Keywords:** Cephalometry, extraction treatment, external apical root resorption, malocclusion, orthodontics

## Abstract

**Objectives:**

To evaluate the cephalometric effects of premolar extraction on skeletal and dental parameters, and on the soft tissues, in patients subsequently treated with fixed appliances. Prevalence and severity of external apical root resorption due to premolar extraction were also examined.

**Materials and methods:**

The dental records of 79 patients treated with fixed appliances were retrieved (groups: extraction, *n* = 19; non-extraction, *n* = 60). Pre- and post-treatment statuses of skeletal, dentoalveolar, and soft tissue variables were analyzed on lateral cephalograms to determine change. Periapical radiographs of the maxillary incisors were assessed for external apical root resorption using the Levander & Malmgren index. The *t*-test, Mann-Whitney *U* test, chi-squared test, and Kruskal-Wallis test were used to analyze the data. Significance was set at *p* < .05.

**Results:**

Changes in the protrusion and proclination of the incisors and in lip position were significantly different between the groups. Prevalence of external apical root resorption in the two groups was similar.

**Conclusions:**

Our findings suggest that extraction therapy affects dentoalveolar traits but not jaw position, nor the risk of root resorption, in patients treated with fixed appliances

## Introduction

The most common malocclusions in Swedish children and adolescents are Angle Class I or Class II malocclusion with dental crowding [1]. An increased overjet, proclined maxillary incisors, and larger overbite are commonly comorbid with a Class II malocclusion, while dental crowding and increased overjet are commonly comorbid with both Class I and II malocclusions [2]. Since these malocclusions can occur due to dental or skeletal discrepancies, or a combination of both, varying treatment strategies are required. To correct malocclusions in adolescents, treatment with fixed orthodontic appliances in combination with the extraction of permanent teeth is commonplace. The protocol for extraction therapy varies depending on type of occlusion, function, and tooth morphology. The teeth most commonly extracted are the first premolars [3].

In studies of orthodontic treatment effects on the antero-posterior and vertical dimensions of the jaws, results have long been controversial, with authors reporting contradicting results in the horizontal and vertical changes that occur during treatment [4–7]. Studies evaluating these effects on jaw rotation report a similar problem: some demonstrate an anterior rotation of the mandible [4,8], while other studies observe no rotational changes [5,9–12]. Contradicting results on the vertical effects of premolar extraction also occur. Some studies report no differences between extraction and non-extraction groups [9,11,13] while others have found an increase in the lower anterior face height and backward rotation of the mandible in patients who had not received extraction treatment [14,15].

An unwanted side effect during orthodontic treatment with fixed appliances is external apical root resorption (EARR). The prevalence of EARR ranges from 65.6% to 98.1% depending on the reported severity and method of assessment [16,17]. Amounts of root resorption vary among patients, and in most cases, loss of root substance is minor [17–20]. Only in a small percentage of cases is resorption more than one-third of the original root length [16,21]. Most affected are the roots of the maxillary incisors [16,22]. Even though EARR is a common side effect of orthodontic tooth movement and extensively investigated, it is difficult to find conclusive evidence on what factors increase the risk of developing EARR [16,17,20,23–27]. Regarding the effects of extraction treatment on the frequency and severity of EARR, the results have also been contradictory [23,28–30]. Evidence for which factors increase the risk of EARR, however, remains weak [23,24,27,31].

Thus, the objectives of this study were, using retrieved lateral cephalograms, to evaluate skeletal, dental, and soft tissue changes over the course of fixed orthodontic appliance treatment carried out at the Department of Orthodontics at the Faculty of Odontology, Malmö University, Malmö, Sweden, that did or did not include premolar extractions. The hypothesis was that the end results of orthodontic treatment differed significantly between the extraction and non-extraction groups. Additional objectives were to assess the prevalence and severity of EARR in the early stages of orthodontic treatment, when a routine assessment of the risk of EARR is normally performed and determine whether patients treated at a university clinic who had received premolar extraction therapy developed EARR to a higher extent than non-extraction patients. The null hypothesis was that premolar extraction increased the risk of EARR.

## Materials and methods

The Swedish Ethical Review Authority approved this study (Dnr 2021-04958).

Dental records were retrieved from the Department of Orthodontics at the Faculty of Odontology, Malmö University, Malmö, Sweden, for all patients treated between 01 January 2010 and 31 December 2020 . All lateral cephalograms and periapical images were exposed by licensed staff at the Department of Oral Radiology, Malmö University.

The following inclusion and exclusion criteria were used to select eligible patients. Eligibility was determined from the dental records.

### Inclusion criteria

- Patients with Angle Class I or Class II malocclusion, moderate-to-severe crowding, and orthodontic treatment with fixed appliances in both jaws. Type of vertical malocclusion did not restrict eligibility.
- Lateral cephalograms at two points during therapy: before treatment start (T_0_), that is, within 3 months before start of treatment; and upon completion of active treatment (T_1_), that is, when the orthodontists judged treatment to be complete. No more than 1 month after the fixed appliances were removed.
- Digital periapical radiographs of the maxillary incisors at two points during therapy: before treatment start (T_0_), that is, within 3 months before start of treatment; and after 6–12 months of active orthodontic treatment, when aligning of the teeth and elimination of any extraction spaces was complete (T_½_). The radiographs were required to have been exposed at the department of Oral Radiology and be both isometric and orthoradial.

### Exclusion criteria

- Previous trauma to the maxillary incisors
- Agenesia of the maxillary incisors
- Dental implants
- Age > 19 years
- Any known syndromes

### Patients

Based on the dental records, 79 patients between 01 January 2010 and 31 December 2020 were eligible. Sixty patients received non-extraction therapy and 19, extraction therapy. In this second group, extracted teeth varied: the four first premo-lars (*n* = 9); the two first premolars in the maxilla and either one second and one first premolar, or two second premolars in the mandible (*n* = 6); or only the two first premolars in the maxillae (*n* = 4). The cephalometric analysis included only patients who had had four premolars extracted (i.e. 15/19).

The root resorption analysis comprised 133 maxillary incisors in 34 patients: 24 patients in the non-extraction group and 10 in the extraction group.

Descriptive data included pre-treatment age and type of malocclusion, type of functional appliance, and treatment time.

### Radiological examination

**Figure 1:**
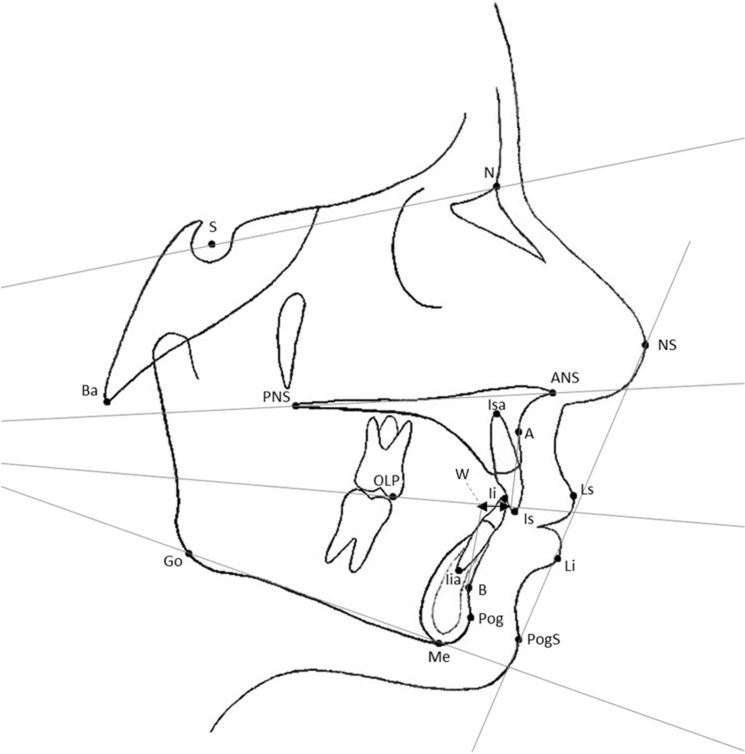
S: Sella; N: Nasion; PNS: Spina Nasalis posterior; ANS: Spina Nasalis anterior; A: Subspinale; isa: incisive superior apex; is: incisive superior; Ii: incisive inferior; iia: incisive inferior apex; B: supramentale; Pog: pogonion; me: menton; (o: gonion; Ba: basion; OLP: occlusal line; posterior point; NS: nasion soft; PogS: pogonion soft; Ls: lip superior, Li: lip inferior; W: wits appraisal.

**Figure 2:**
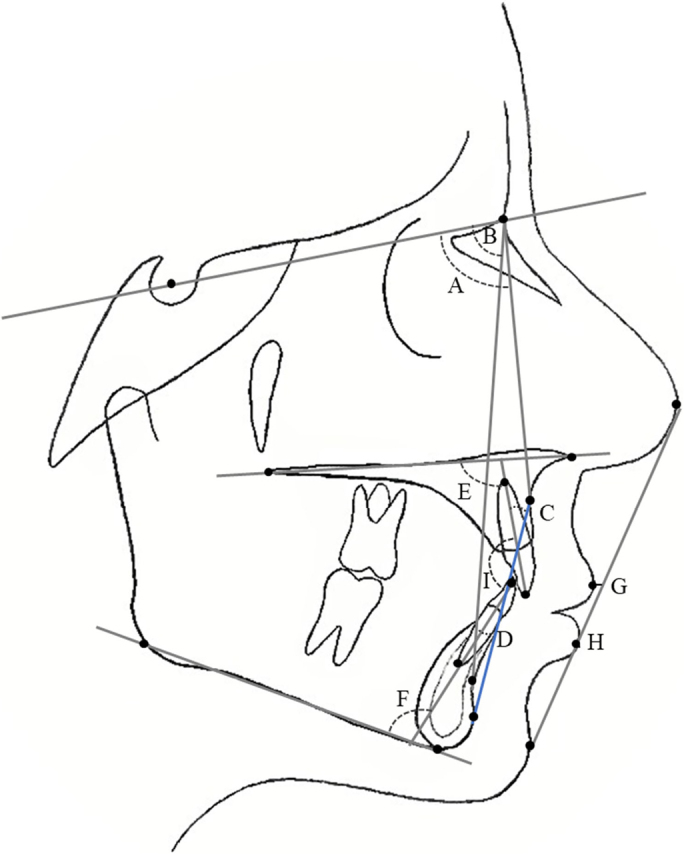
A: SNA angle; B: SNB angle; C: incisor superior/A-Pog angle; D: incisor inferior/A-Pog angle; E: incisor superior/Nasal plane angle; F: incisor inferior/mandibular plane angle; G: labrale superior to NS-PogS distance; H: labrale inferior to NS-PogS distance; I: interincisal angle.

All lateral cephalograms (taken at T_0_ and at T_1_) were traced using FACAD® Orthodontic Tracing Software, version 3.12 (ILEXIS AB, Bielkegatan 1A, SE-58221 Linköping, Sweden). The cephalo-grams had been taken with one of two radiographic units: a Morita Veraviewepocs 3D X-550 or a Morita 3De-CP. The linear measurement tool in FACAD was used to calibrate the cephalo-grams and eliminate differences in magnification. Tracings were made with reference to 15 hard-tissue and four soft-tissue landmarks. The analysis comprised 13 angular and 5 linear measurements based on the Bergen analysis (Figures 1 and 2).

All periapical radiographs (taken at T_0_ and at T_½_) were evaluated for incidence and severity of root resorption of the maxillary incisors before and during treatment. All radiographs were assessed with Planmeca Romexis^®^ imaging software (Planmeca Oy, Asentajankatu 6, FI-00880 Helsinki, Finland). Radiographs taken at T_½_ were compared with the radiographs at T_0_. The quantity of EARR was assessed according to the scoring system of Levander & Malmgren [32]. Scores ranged from 0 to 4 (grade 0: no visible root resorption; grade 1: irregular root contour; grade 2: minor resorption ≤ 2 mm; grade 3: severe resorption from 2 mm to 1/3 of the original root length; and grade 4: extreme resorption > 1/3 of the original root length).

One observer (ME) evaluated all radiologic data on a Barco^®^ display (Barco Coronis Fusion cor c f 6MP led 30”1h1Fx00, Barco Beneluxpark 21, 8500 Kortrijk Belgium) calibrated for x-ray diagnostics; ambient luminance never exceeded 50 lux. Analysis of the periapical radiographs was blinded, and of the tracings, partially blinded; premolar extraction was visible on the post-treatment cephalograms.

### Inter- and intra-observer reliability

Inter-observer calibration comprised tracing 20 randomly selected lateral cephalograms. Four observers were calibrated: one resident orthodontist (8 years of experience), one general practitioner at the Department of Oral Radiology (11 years), and two orthodontic assistants (3 and 21 years). During calculations of inter-observer reliability, discrepancies were discussed until a consensus on landmark placement was reached.

Intra-observer agreement in the cephalometric study was determined by randomly selecting 20 records (two tracings per record: the pre- and the post-treatment cephalogram tracing) and re-evaluating the tracings (*n* = 40) 3 months after the first assessment.

Intra-observer agreement in the assessment of root resorption was determined by randomly selecting 16 patients and re-evaluating the maxillary incisors 3 months after the first assessment.

### Statistical analysis

IBM SPSS (Version 28.0.1.1 [14]) was used in all statistical analysis of the data; significance was set at *p* < .05.

Because the patient groups were not homogenous, non-parametric statistical tests were used.

Between-group comparisons of skeletal, dental, and soft tissue measurements were calculated using the Mann-Whitney *U* test. The independent samples *t*-test, the Mann-Whitney *U* test, and the Kruskal-Wallis test were used to test for potential confounding factors. Differences between type of malocclusion, gender, and type of functional appliance were assessed.

EARR frequencies in the maxillary incisors were compared between the non-extraction and extraction groups using the chi-squared test. Due to the varying types of functional appliances and malocclusions in this study, we used the chi-squared test to assess if these were confounding factors for the degree of EARR.

Again, because the patient groups were not homogenous, analyzes were made using non-parametric statistical tests. We used the Kruskal-Wallis test to analyze correlations between degree of root resorption at T_½_ and the following measurements at T_0_: the ILs/NL angle, the ILs/APog angle, the Is-toAPog distance, overjet, and age.

Intra-class correlation coefficients (ICC) were calculated to identify both inter- and intra-observer reliability in the cephalometric analysis. Cohen’s weighted kappa was used to identify intra-observer reliability in the analysis of EARR.

No sample size calculation was made due to the cross-sectional nature of the study.

## Results

Records of the 79 eligible patients (mean age 14.4 years, range 11.2–18.8) were retrieved. (Table 1). All subjects received bimaxillary fixed appliances (0.022 slot size, MBT prescription, Victory Series, 3 M Unitek, Monrovia, California, USA) and, when applicable, Class II elastics or fixed functional appliances. Average treatment time and gender distribution were similar between the malocclusion groups (Table 1).

**Table 1:** Descriptive data of the patient Cohort (*n* = 79).

Sagittal classification of malocclusion	Number (n)	Percent (%)	Gender (M/F)	Treatment time (mean y)	No. of cases	
					FFA or ClII el	Extraction
Angle class I	30	38.0	13/17	1.75 ± 0.62	2 FFA	10 Ex.4
					15 ClII el	
Angle class II:1	36	45.6	13/23	1.97 ± 0.72	12 FFA	4 Ex.4
					22 ClII el	4 Ex.2
Angle class II:2	13	16.5	5/8	1.95 ± 0.62	1 FFA	1 Ex.4
					12 ClII el	
Total	79	100	31/48	1.9 ± 0.67	15 FFA	15 Ex.4
					49 ClII el	4 Ex.2

FFA: Fixed functional appliances; ClII el: Class II elastics; Ex.4: Extraction of four premolars (maxillary and mandibular); Ex.2: Extraction of two premolars (maxillary).

### Cephalometric analysis

Before treatment (T_0_), the non-extraction and extraction groups differed significantly only in the ML/SNL angle, the ML/NL angle, and the Ii-to-APog distance (*p* < .05, Table 4). At the end of treatment (T_1_), the differences between groups in the ML/SNL angle and the NL/ML angle remained significant (*p* < .05). Differences between the groups at T_1_ were significant in all variables relating to the position and inclination of the maxillary and mandibular incisors as well as the Wits appraisal (Table 2).

**Table 2:** Comparisons of measurement differences on digital radiographs between the non-extraction (*n* = 60) and the extraction (*n* = 15) groups before and after treatment.

Measurements on radiographs										
	Before treatment (T_0_)					After treatment (T_1_)				
	Non-extraction		Extraction		*p*-value	Non-extraction		Extraction		*p*-value
	Median	Mean	Median	Mean		Median	Mean	Median	Mean	
SNA^a^	82.45	82.09 ± 3.58	80.4	81.12 ± 2.65	.257	81.4	81.42 ± 3.55	80.6	80.21 ± 3.27	.192
SNB^a^	78.1	77.34 ± 3.33	76.0	76.51 ± 2.86	.249	78	77.42 ± 3.45	76.9	76.55 ± 2.75	.228
ANB^a^	4.7	4.74 ± 2.34	4.3	4.61 ± 2.62	.726	3.6	4.0 ± 2.33	3.9	3.67 ± 2.79	.837
ANPog^a^	2.95	3.66 ± 2.98	4.2	4.17 ± 3.08	.538	2.5	2.85 ± 3.06	3.0	2.97 ± 3.52	.648
ML/NSL^a^	32.65	33.02 ± 6.15	36.6	37.18 ± 4.88	.014*	33.4	33.38 ± 6.64	37.1	37.17 ± 4.40	.021*
NL/NSL^a^	6.95	7.30 ± 3.51	6.6	6.95 ± 3.52	.569	7.05	7.79 ± 3.59	7.4	7.41 ± 3.56	.721
ML/NL^a^	25.5	25.73 ± 5.73	30.1	30.23 ± 6.43	.009*	25.5	25.59 ± 6.19	29.5	29.76 ± 5.99	.019*
ILs/NL^a^	111.4	110.86 ± 10.52	112.3	115.28 ± 10.13	.286	115.25	114.51 ± 6.85	108.5	108.47 ± 6.96	.005*
ILi/ML^a^	94.65	95.33 ± 6.5	95.6	95.12 ± 8.50	.942	103.1	102.65 ± 7.3	90.9	92.04 ± 5.59	< .001*
ILs/APog^a^	29.9	29.16 ± 11.17	30.9	35.79 ± 12.59	.192	30.8	31.28 ± 5.79	25.9	27.09 ± 6.85	.009*
ILi/APog^a^	22.1	22.73 ± 4.96	25.2	24.83 ± 7.06	.301	31.55	31.48 ± 5.28	24.1	23.19 ± 4.34	< .001*
Wits^b^	2.5	2.42 ± 3.1	1.0	1.49 ± 4.03	.213	0.55	0.43 ± 2.12	−0.7	−0.85 ± 2.28	.041*
SNPog^a^	78.9	78.44 ± 3.49	76.7	76.94 ± 2.97	.080	79.25	78.58 ± 3.73	77.0	77.24 ± 2.81	.116
Is-to-Apog^b^	6.65	5.99 ± 3.53	5.8	7.53 ± 4.28	.263	5.9	6.3 ± 2.47	3.4	4.09 ± 2.39	< .001*
Ii-to-Apog^b^	0.8	0.64 ± 2.67	2.0	2.85 ± 3.13	.029*	3.4	3.62 ± 2.38	0.6	1.53 ± 2.61	.001*
Interincisal^a^	125.65	128.11 ± 14.09	123.3	119.37 ± 17.67	.190	116.2	117.24 ± 7.99	129.9	129.72 ± 9.47	< .001*
Ls-EL^b^	−2.8	−2.8 ± 2.87	−2.1	−1.64 ± 3.86	.491	−3.4	−3.44 ± 2.81	−4.7	−3.98 ± 3.78	.333
Li-EL^b^	−1.65	−1.42 ± 3.01	−1.3	0.18 ± 4.23	.272	−0.95	−0.47 ± 3.38	−2.0	−1.48 ± 4.18	.181

T_0_: Pre-treatment (radiographs taken within 3 months before treatment start); T_1_: Post-treatment (radiographs taken within 1 month following treatment end).

* statistical significance (*p*<.05).

a angle(°).

b distance(mm).

**Table 3:** Median and mean within-group differences in measurements on digital radiographs taken before (T_0_) and after (T_1_) orthodontic treatment in the non-extraction (*n* = 60) and the extraction (*n* = 15) groups, and between-group significance.

Treatment group		Median	Mean difference	SD	Significance
SNA^a^	Non-extraction	−1.8	−0.7	1.2	>.05
	Extraction	−1.0	−0.9	1.2	
SNB^a^	Non-extraction	0.15	0.1	1.1	>.05
	Extraction	−0.1	0.04	1.0	
ANB^a^	Non-extraction	−0.65	−0.7	1.3	>.05
	Extraction	−0.8	−0.9	1.3	
ANPog^a^	Non-extraction	−0.8	−0.8	1.2	>.05
	Extraction	−1.0	−1.2	1.5	
ML/NSL^a^	Non-extraction	0.3	0.4	1.6	>.05
	Extraction	0.3	0.0	1.2	
NL/NSL^a^	Non-extraction	0.5	0.5	1.1	>.05
	Extraction	0.3	0.5	1.3	
ML/NL^a^	Non-extraction	0.2	−0.1	1.7	>.05
	Extraction	−0.2	−0.5	1.5	
ILs/NL^a^	Non-extraction	3.6	3.7	10.8	.004*
	Extraction	−3.3	−6.8	10.3	
ILi/ML^a^	Non-extraction	6.6	7.3	6.5	<.001*
	Extraction	−1.6	−3.1	4.9	
ILs/APog^a^	Non-extraction	2.55	2.1	10.4	.002*
	Extraction	−5.6	−8.7	9.9	
ILi/APog^a^	Non-extraction	7.5	8.7	6.7	<.001*
	Extraction	−0.1	−1.6	4.8	
Wits^b^	Non-extraction	−1.7	−2.0	2.5	>.05
	Extraction	−2.9	−2.3	2.8	
SNPog^a^	Non-extraction	−0.05	0.1	1.1	>.05
	Extraction	0.4	0.3	0.8	
Is to APog^b^	Non-extraction	0.4	0.3	2.6	<.001*
	Extraction	−3.4	−3.7	3.3	
Ii to APog^b^	Non-extraction	3.3	3.0	1.8	<.001*
	Extraction	−1.3	−1.3	1.1	
Interinc.^a^	Non-extraction	−10.5	−10.7	13.4	<.001*
	Extraction	7.1	10.4	11.5	
Ls to EL^b^	Non-extraction	−0.65	−0.6	1.9	.014*
	Extraktion	−1.8	−2.3	2.3	
Li to EL^b^	Non-extraction	1.0	0.9	2.0	<.001*
	Extraction	−1.2	−1.7	1.8	

T_0_: Pre-treatment (radiographs taken within 3 months before treatment start); T_1_: Post-treatment (radiographs taken within 1 month following treatment end).

* statistical significance (*p*<.05).

a Angle (°).

b Distance (mm).

**Table 4:** Correlation between measurements at T_0_ and degree of root resorption at T_½_.

Variable	Range	*p*-value
Age at T_0_	11.2y–18.3y	.598
ILs/NL angle	88.6°–135.7°	.030*
ILs/APog angle	6.2°–52.7°	.147
Is-to-Apog distance	−0.8 mm–15.0 mm	.273
Overjet	1.0 mm–15.0 mm	.809

T_0_: Pre-treatment (cephalograms taken within 3 months before treatment start); T_½_: Mid-treatment (when aligning of the teeth and elimination of any extraction spaces was complete);

* Significance set at *p* < .05; Independent-samples Kruskal-Wallis test.

Comparisons of the changes occurring during orthodontic treatment found a significant difference between the groups in all variables relating to the position and inclination of the incisors. The ILs/NL angle, the ILs/APog angle, the ILi/ML angle, and the ILi/APog angle increased in non-extraction patients, whereas these variables decreased in the extraction patients (*p* < .01). The Is-to-APog and Ii-to-APog distances increased in the non-extraction patients and decreased in the extraction patients (*p* < .001, Table 3).

When we controlled for Class II division 2 malocclusion, the ILs/APog angle (*p* = .018) and the Is-to-APog distance (*p* < .001) also decreased in the non-extraction group, although significantly less compared to the decrease in the extraction group. The ILs/NL angle remained mostly unchanged in the non-extraction but decreased in the extraction group (*p* = .025).

The changes in Ls-EL distance also differed between groups, decreasing in all patients, and significantly more so in the extraction group (*p* = .014). However, when the Class II division 2 patients were controlled for, this difference was not significant (*p* > .05). The Li-EL distance decreased in the extraction group but increased in the non-extraction group (*p* < .001, Table 3).

Comparisons of patients who received only bimaxillary fixed appliances with patients whose treatment included Class II elastics or fixed functional appliances only found differences in the SNB angle and the Wits appraisal. The SNB angle increased in the patients who used Class II elastics or a Herbst appliance, compared to those who received only bimaxillary fixed appliances (*p* = .046). The Wits appraisal decreased significantly in patients who used Class II elastics, a Herbst appliance, or a Forsus appliance, compared to those who received only bimaxillary fixed appliances (*p* < .001).

No significant between-group change was found in jaw position or rotation (Table 3).

### External apical root resorption

The dental records of 34 patients included intraoral periapical radiographs of the maxillary incisors at T_0_ and at T_½_. At T_0_, none of the 34 patients showed any signs of root resorption. At T_½_, EARR was found in 64.7% of the patients and on 47.4% of the 133 assessed maxillary incisors.

Comparisons of the cephalometric variables related to the position of the maxillary incisors and age at T_0_ with the frequency and degree of EARR at T_½_ found a significant tendency for patients with a smaller ILs/NL angle before orthodontic treatment to have a higher degree of EARR at T_½_ (*p* = .030). No other significant correlations were found (Table 4).

No significant difference in frequency or degree of EARR was found between the two extraction groups (*p* > .05) or between patients who had received only bimaxillary fixed appliances and patients treated with Class II elastics or fixed functional appliances (*p* > .05).

### Intra-observer reliability

Intra-observer reliability for the cephalometric measurements (ICC) was 0.95–0.98 for all measured variables. Cohen’s weighted kappa for intra-observer reliability in the analysis of EARR was 0.448 on the patient level and 0.547 on the tooth level.

## Discussion

This study investigated the cephalometric effects of premolar extraction on skeletal and dental parameters, and on the soft tissues, in adolescent patients with Angle Class I or Class II malocclusions and dental crowding during orthodontic treatment with fixed appliances. We found that the incisors in both jaws became significantly more retroclined and retruded in patients who had received extraction therapy before orthodontic treatment compared with those who had not.

### Soft tissue changes

The soft tissue measurements in this study comprised the positions of the upper and lower lip in relation to the E-line. During treatment, the lower lip was slightly protruded in non-extraction patients and retruded in extraction patients (*p* < .001). Although the upper lip was retruded in all patients during treatment, retrusion was significantly more pronounced in the extraction group (*p* = .014). These results were comparable to previous studies [33,34].

In contrast, Weyrich & Lisson [35] observed a retrusion of both lower and upper lips after orthodontic treatment, with no significant differences between patients who had received extraction therapy and those who had not. Basciftci & Usumez [36] examined patients treated with extraction or non-extraction therapy; in Class I subjects, they found differences between the treatment groups that were comparable to what we found. However, they presented no significant differences between the groups in Class II subjects. Comparisons of patients with Class I and Class II: division 1 malocclusion in the present study found that changes in upper and lower lip position during treatment were comparable.

A retrusive effect on the position of the upper and lower lip has also been reported for the first premolar extractions done only in the maxilla [34].

The effects of natural growth must also be considered when assessing changes in the soft-tissue profile made by measuring the distance from the lips to the E-line. The lips become more retruded with time, and this change occurs mainly between 15 and 25 years of age [37]. The patients in the present study were adolescents (mean age 14.4 years), which means that growth-related changes in the position of the lips could have affected the results. Previous studies have analyzed adolescent [35] and adult [33] patients, which might explain the varied results between these studies and the present study.

Interestingly, comparisons of the non-extraction and extraction groups at T_1_ in this study found no significant differences in the position of the upper and lower lip (*p* > .05) between groups. Our results suggest that the choice of non-extraction or extraction treatment had no effect on soft tissue status.

### Dental changes

All the dental variables we measured changed during orthodontic treatment, and between-group comparisons of the changes were all significant. The degree of retroclination and retrusion of the maxillary incisors was less in the non-extraction group compared to the extraction group. In the non-extraction group, the mandibular incisors were proclined as well as protruded, and in the extraction group, slightly retroclined and retruded. These findings are comparable to previous studies [4,36,38,39].

### Skeletal changes

No significant differences between the non-extraction and the extraction groups were found concerning changes in the antero-posterior position of the jaws, nor were any significant changes in jaw rotation found between the groups. This agrees with some studies [9,11,12], while others have found contrary results, reporting a restrictive effect of extraction therapy on anterior facial height growth and mandibular rotation [6,15].

Comparing studies using linear/angular measurements with those using superimposition is difficult, considering that changes in the ratio of anterior-to-posterior face height do not necessarily reflect the pattern of mandibular basal rotation [40]. Measurements using the mandibular plane are also unreliable since changes in mandibular rotation can be masked by the remodelling of the mandibular border that occurs during growth [41]. A comparison using superimpositions could strengthen the reliability of the analysis.

### Analysis of external apical root resorption

We evaluated EARR on the maxillary incisors [21,22] and found a below-average frequency of EARR among the patients in the present study [16]. This could be due to either the short period of 6–12 months of active treatment at T_½_, or the use of a subjective scoring index [18]. In addition, the small number of patients with intraoral periapical radiographs of the maxillary incisors at T_½_ might have influenced the incidence of EARR, and the actual frequency of resorbed incisors could be higher.

Smale et al. [42] studied the prevalence of EARR after approximately 6 months of treatment. They found resorption of the maxillary incisors in 82.5% of their patients, and in 53% of the maxillary incisors that they analyzed, comparable to our findings of 47.4% of the maxillary incisors. Their study used a scoring index adapted from Levander & Malmgren, with the added benefit that all images had been taken with a standardized paralleling technique and a digital reconstruction technique to adjust for projection errors. Still, they concluded that agreement was low when EARR was scored using this index [42].

Severe root resorption as a result of orthodontic treatment is rare [16,21,43]. In this study, none of the patients sustained severe root resorption. It is possible, though, that the radiologic control was done too soon after treatment started and that the movement of the maxillary incisors were not yet of a magnitude that increased the risk of apical root resorption, since some researchers have found correlations between severe EARR and longer treatment times [18,25]. Recently, however, other researchers have contested this correlation [16,27]. This study found no evidence that extraction of the two first maxillary premolars increased the risk of EARR in the maxillary incisors. This contrasts with previous findings that extraction of these premolars was a risk factor for apical resorption of the maxillary incisors [21,43,44].

The findings in this study suggests that the initial inclination of the central maxillary incisor compared to the palatal plane was correlated with the severity of EARR. Previous studies have shown that horizontal movement of the maxillary incisors, where the apex was moved in a lingual direction, was strongly correlated with a higher risk of root resorption [26,45]. The strongest correlation with EARR was found when the apex of the incisor was both intruded and torqued lingually. However, not all studies agree with these results, and the quality of evidence remains low [27].

Since no lateral cephalograms in this study were available at T_½_, comparisons between the amount of apical torque or horizontal movement of the apex and the prevalence of EARR were not possible.

Also, we found no correlation between the amount of overjet at the start of the treatment and EARR. This result is contrary to previous findings [21].

### Strengths and limitations

The changes that occurred in both the position and inclination of the incisors during orthodontic treatment were similar to those in previous studies comparing non-extraction with extraction treatment; this strengthens the reliability of our findings.

We used the resorption index suggested by Levander & Malmgren, which has the advantage of not being dependent on the standardization of the periapical radiographs [32]. The minor amount of EARR that, feasibly, might have occurred after only 6–12 months of orthodontic treatment would most likely be difficult to detect, and would explain the low agreement in the intra-observer analysis.

Inter-observer reliability in the cephalometric measurements was excellent, with the highest disagreement occurring in the Wits appraisal and the ILi/APog angle. Difficulties in identifying the OLp landmark and mandibular incisor apex in the lateral cephalogram, due to superimposing structures, likely explains this.

When indicated, intraoral radiographs were taken at 6–12 months after treatment start, when correct tooth alignment and elimination of extraction spaces was achieved. This means that treatment was incomplete at T_½_. Teeth with visible signs of EARR at this early stage of treatment could be at a higher risk of developing severe root resorption later [18], which makes the early radiographic evaluation of the incisors valuable in identifying those patients who are more susceptible to severe root resorption.

The patient sample was selected based on the availability of lateral cephalograms for both pre- and post-treatment. Since all patients treated during a 10-year interval who fulfilled the criteria were included, the sample was heterogeneous concerning orthodontic diagnoses, treatment method, and treating orthodontists. Thus, several subgroups with relatively small numbers of patients were created. Follow-up cephalograms were taken only when the orthodontist considered the radiographic examination to be of diagnostic value, according to Swedish national radiation safety regulations [46]. This created a selection bias since our patients were not randomly selected and might not be representative of the average demographic.

There was considerable variation in the age of the patients at treatment start (11.2–18.8 years). This meant that many of our patients were still growing, which might have affected the results [37]. In addition, the number of patients who received extraction treatment differed significantly from those who had not.

This study emphasizes the need for more research on the risk of EARR in adolescents undergoing orthodontic treatment.

## Conclusions

The results of this study suggest that the incisors in both jaws become less protruded and less proclined when bimaxillary premolar extractions are performed at the start of orthodontic treatment compared with when premolars are not extracted.

Extraction had a significant effect on the changes of lip position in relation to the E-line compared to non-extraction. However, patients in both groups had similar soft tissue values after orthodontic treatment.

Extraction therapy was not correlated with a higher risk of EARR, but incisors with a smaller ILs/NL angle at the start of orthodontic treatment could be at an increased risk of developing EARR.

## Data Availability

The data that support the findings of this study can be obtained from the corresponding author [ME] upon reasonable request.
